# A machine learning model and molecular clusters of epigenetic chromatin regulators in tuberculosis based on bioinformatics and clinical samples

**DOI:** 10.1038/s41598-025-25858-9

**Published:** 2025-11-25

**Authors:** Huawei He, Liuying Wei, Lanwei Nong, Beibei Gong, Chaoyan Xu, Qingdong Zhu

**Affiliations:** 1 Department of Tuberculosis, The Fourth People’s Hospital of Nanning, Nanning, China; 2 Infectious Disease Laboratory, The Fourth People’s Hospital of Nanning, Nanning, China; 3 Department of Nursing, The Fourth People’s Hospital of Nanning, Nanning, China

**Keywords:** Tuberculosis, Epigenetics, Chromatin regulators, Immune cell infiltration, Machine learning, Diagnostic markers, Predictive markers, Bacterial infection

## Abstract

The role of chromatin regulators (CRs) in mediating epigenetic changes during tuberculosis (TB) infection remains poorly understood. This study aimed to determine the efficacy of CRs in diagnosing TB and characterizing its heterogeneity. GSE83456 dataset was analyzed to identify differentially expressed CRs (DE-CRs) and immune cell infiltration in patients with TB. Consensus clustering was used to classify patients with TB based on DE-CR expression patterns. The optimal machine learning model was selected from four algorithms (Random Forest (RF), Support Vector Machine (SVM), Generalized Linear Model (GLM), and eXtreme Gradient Boosting (XGB)) to differentiate between the molecular clusters. Validation was performed using an external dataset (GSE152532). Blood samples were collected from healthy individuals and patients with pulmonary TB (PTB) or tuberculous meningitis (TBM). Analysis identified 15 DE-CRs, which were used to stratify patients with TB into two distinct molecular clusters exhibiting divergent immune microenvironment characteristics. The XGB model exhibited superior performance in distinguishing these clusters (area under the receiver operating characteristic curve = 0.965). From this model, a five-gene signature (*DHRS9*, *HIST1H2BK*, *C16orf74*, *SLC30A1*, and *GBP1*) was identified. This signature effectively predicted TB subtypes and was significantly associated with active TB (ATB) in an external validation set. Clinically, *IFIT3* expression was validated as being significantly elevated in the blood of patients with TB (including PTB and TBM) compared to healthy controls, thereby confirming its potential role as a pan-TB biomarker. Our study revealed that CRs are closely associated with immune infiltration and heterogeneity in TB. We developed a robust XGBoost model based on a five-gene signature for accurate TB subtyping and disease-status assessment. Elevated IFIT3 expression underscores the value of CRs as novel biomarkers for TB diagnosis.

**Supplementary Information:**

The online version contains supplementary material available at 10.1038/s41598-025-25858-9.

## Introduction

 Tuberculosis (TB), caused by *Mycobacterium tuberculosis* (*M.tb.*), remains a significant global health burden, with an estimated 10 million new cases annually^1,2^. The limitations of current diagnostic methods, including the low positivity rate of culture-based detection^3–6^ and particular challenges in diagnosing extrapulmonary TB (EPTB)^7,8^, highlight the urgent need for novel biomarkers.

This has resulted in the exploration of host epigenetics^9–12^, which is increasingly recognized as pivotal in *M.tb*.-host interactions^13,14^. *M.tb.* infection can alter host histone modifications to modulate immune responses^13–15^. These modifications are governed upstream by chromatin regulators (CRs), which are proteins including histone modifiers, chromatin remodelers, and DNA methylators that act as writers, readers, and erasers of epigenetic marks. They regulate chromatin architecture and gene expression in response to infection^16–18^. The roles of specific CRs^15^ and DNA methylation patterns^19^ in TB have been extensively reported; however, the existing literature is fragmented. A systematic comprehension of the global CR landscape in TB, its collective impact on immunoregulation, and its diagnostic potential remains elusive.

Therefore, our study aims to comprehensively investigate CRs in TB through an integrated bioinformatics and machine learning (ML) approach. This strategy is supported by successful precedents in other diseases, including the development of a random forest (RF) model based on ferroptosis-related genes for the early diagnosis of acute myocardial infarction^20^. We identified differentially expressed CRs (DE-CRs) and investigated their association with immune cell infiltration. Our objective was to classify patients with TB into molecular subtypes based on CR expression and to construct an ML model for discriminating TB subtypes and disease states (latent versus active). Finally, we clinically validated key CR markers in patient blood samples. Our findings provide novel insights into the epigenetic regulation of TB and establish a foundation for utilizing CRs as biomarkers for improved diagnosis and patient classification.

## Materials and methods

### Experimental design

**Fig. 1:**
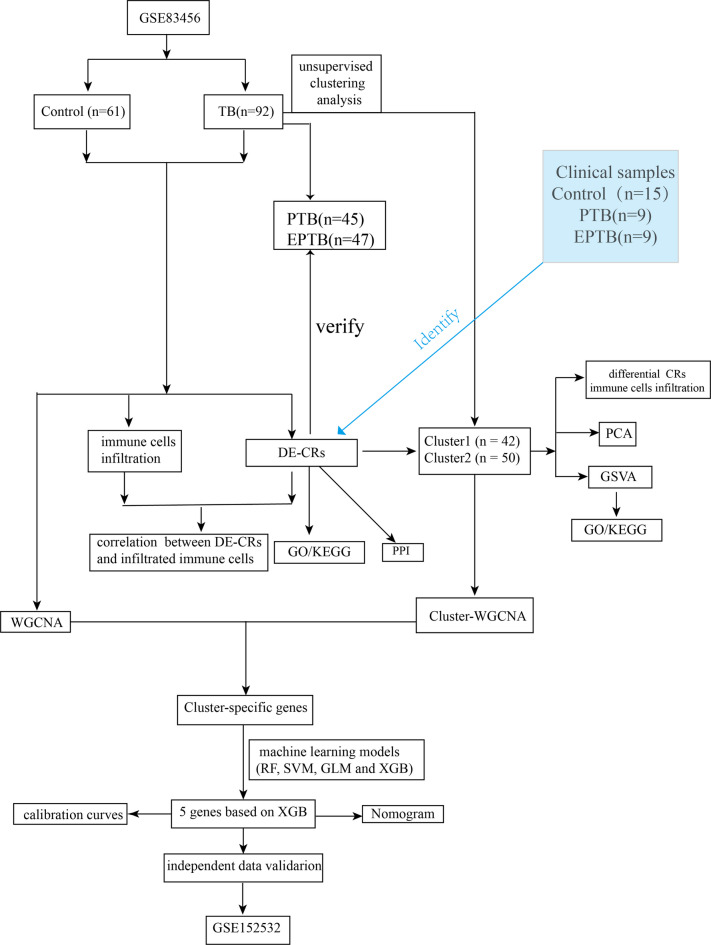
Flow diagram of the experiment.

The experimental design is presented in Fig. 1.

### Data acquisition and preprocessing

Gene expression datasets GSE83456 and GSE152532, which include data from both healthy donors and individuals with TB, were downloaded from the Gene Expression Omnibus (GEO) repository. Data preprocessing was performed using Perl. GSE83456 dataset (GPL10058 platform), including 61 healthy controls and 92 individuals with TB (after excluding individuals with sarcoidosis), was used to identify DE-CRs and construct an ML model. GSE152532 dataset (GPL10058 platform), containing 11 healthy controls and 136 individuals with TB, was used as an external validation set to evaluate the accuracy of the developed model. We obtained CRs (870 genes) from a study by Lu et al^18^.. R programming language (version 4.1.3) was used for data analysis.

### Identification of DE-CRs in TB

Volcano plots were constructed using the “limma” R package to identify differentially expressed genes (DEGs) in GSE83456 dataset. DEGs were identified using an adjusted *p*-value (adj.P.Val) threshold of < 0.05, controlling for the false discovery rate using the Benjamini-Hochberg procedure. The intersection genes of DEGs and CRs were identified using “Venn” R package and labeled DE-CRs. Protein-protein interaction (PPI) analysis of DE-CRs was performed using the STRING website (https://cn.string-db.org/). The “ggpubr” and the “pheatmap” R packages were used to generate the box plot and heatmap of DE-CRs, respectively. The “corrplot” R package was used to correlate DE-CRs and investigate the relationships between genes.

### Comprehensive analysis of DE-CRs

Gene ontology (GO) functional enrichment analysis comprises three fundamental terms: biological process, cellular component, and molecular function (MF). Kyoto encyclopedia of genes and genomes (KEGG) enrichment analysis primarily focuses on enriched pathways^21,22^. We conducted GO and KEGG analyses to comprehensively evaluate MFs of DE-CRs.

### Correlation analysis between CRs and infiltrated immune cells

To characterize immune cell infiltration from gene expression data, we used CIBERSORT algorithm^23^. This computational approach simplifies transcriptomic data to estimate the relative fractions of 22 immune cell types, thereby overcoming the potential limitations of technical variability associated with flow cytometry^24^. CIBERSORT has been extensively employed to prevent inaccurate results caused by cell loss and damage. CIBERSORT algorithm (https://CIBERSORT.stanford.edu/) was used with the LM22 eigenmatrix to determine the proportional representation of 22 distinct immune cell types within each specimen based on gene expression data obtained from GSE83456 dataset^25^. The 22 distinct immune cells included memory B cells, naïve B cells, activated dendritic cells, resting dendritic cells, eosinophils, M0-macrophages, M1-macrophages, M2-macrophages, activated mast cells, resting mast cells, monocytes, neutrophils, activated NK cells, resting NK cells, plasma cells, activated memory CD4 T cells, resting memory CD4 T cells, naïve CD4 T cells, CD8 T cells, follicular helper T cells, and gamma delta T cells. We conducted a correlation coefficient analysis to elucidate the association between DE-CRs and relevant characteristics of immune cells. A statistically significant association is indicated by a probability (*p*)-value *<* 0.05 when Spearman’s correlation coefficient is employed. R package “corrplot” was used to present the results. Spearman’s correlation analysis was performed between the 15 DE-CRs and 22 immune cell types. Due to the large number of correlation tests performed (*n* = 330), we acknowledge that the use of nominal *p*-values (*p* < 0.05) without multiple testing correction increases the risk of false positive findings. However, our findings were reported using nominal significance levels, as this analysis was exploratory and hypothesis-generating, designed to identify potential relationships for future research.

### Clustering of individuals with TB

We classified 92 individuals with TB into different subgroups based on the expression profiles of DE-CRs using the “ConsensusClusterPlus” R package for unsupervised clustering analysis. We defined k values between 1 and 9, generated different subtypes, and determined the ideal number of clusters using consensus scores. Principal component analysis (PCA) was used to demonstrate the distribution of the subtypes.

### Gene set variation analysis (GSVA)

GSVA was conducted using “GSVA” R package to determine the differences in enriched gene sets across different CR clusters. The gene sets “c5.go. symbols” and “c2.cp. kegg. symbols” were obtained from the Molecular Signature database (MSigDB) for use in GSVA. The analysis used “limma” R package (version 4.13) to identify the differential expression pathways and biological activities by comparing GSVA scores across multiple clusters of CRs. A statistically significant difference was defined at *p*-value < 0.05.

### Weighted gene co-expression network analysis (WGCNA)

“WGCNA” R package was used for the WGCNA authentication co-expression module. To improve the reliability and precision of the results from WGCNA, only the top 25% of genes exhibiting the highest variance were selected for analysis. A weighted adjacency matrix was generated to determine the most efficient form of soft power. The matrix underwent a subsequent transformation, yielding the topological overlap matrix (TOM). Based on the hierarchical clustering tree algorithm, the researchers implemented the TOM dissimilarity measure, specifically the 1-TOM variant. This was used to determine the module, with a minimum module size threshold of 100. Every module was assigned a color at random. The genes within the module signature represented the overall gene expression pattern observed within each module. Module significance indicated the correlation between modules and disease conditions. Gene significance was used to quantify the degree of the association between a specific gene and a clinical phenotype.

### Construction and validation of a nomogram model

Nomogram models were built to evaluate TB clusters using the “rms” R package. Each predictor was assigned a score, and the “total score” was calculated as the sum of all predictor scores. The predictive capability of the nomogram model was evaluated using calibration curves and decision curve analysis (DCA).

### Construction of a predictive model based on multiple ML methods

ML is considered a subset of artificial intelligence involving algorithms that could define their own rules from input data through iterative training and improvement, without explicit human programming^26^. The four ML models, including random forest (RF), support vector machine (SVM), generalized linear model (GLM), and eXtreme gradient boosting (XGB), have been extensively used in the prediction of multiple diseases, including infectious diseases^27–31^. The comparative evaluation of multiple classifiers, as used in our study, is a well-established method for identifying the optimal predictive model for a given dataset. This methodology has demonstrated exceptional efficacy in addressing comparable biomedical classification challenges^32^. The dataset was randomly divided into a training set (70%) and an independent test set (30%) using a random seed of 42 to ensure reproducibility. The “caret” R package was used to construct ML models, specifically RF, SVM, GLM, and XGB. The hyperparameters of each ML model were based on our previous study^33^: RF: ntree: 500, mtry: 3, nodesize: 1; SVM: C: 1, sigma: caret, prob. model: true; GLM: family: binomial; XGB: nrounds: 150, maxdepth: 6, eta: 0.3, gamma: 0, subsample: 1, colsample_bytree: 1, and lambda: 1. These models were constructed using two distinct CR clusters. The “pROC” R package was used to visualize the area under the receiver operating characteristic (ROC) curve. Consequently, ML model that was most appropriate for the research objectives was identified. Five genes with the highest significance were selected as the major predictor genes associated with TB. To validate the diagnostic model, an ROC curve analysis was conducted on the GSE152532 dataset. Subsequently, a correlation analysis was performed on the clinical characteristics of 136 individuals with TB from GSE152532 dataset using the key predictive genes of TB.

### Identification of DE-CRs in individuals with TB

We collected 15 blood samples from healthy individuals (control group), nine from individuals with pulmonary TB (PTB group), and nine from individuals with tuberculous meningitis (TBM; EPTB group). All procedures involving human blood samples from patients with TB were conducted in a Biosafety Level 2 laboratory, following the guidelines from the Chinese Center for Disease Control and Prevention. Standard operating procedures and personal protective equipment (including lab coats, gloves, and safety goggles) were strictly employed to ensure the safety of personnel and prevent environmental contamination. The 18 individuals with TB were included in the TB group. Blood samples were collected from individuals with TB before they received anti-TB therapy. The exclusion criteria were as follows: malignant tumor, metabolic disorders, non-B infectious disease, and severe impairment of consciousness. Informed consent was obtained from individuals or their guardians. This study adhered to the principles of the Declaration of Helsinki. The Fourth People’s Hospital of Nanning’s institutional review board granted ethical approval. The primers of DE-CRs were as follows: *IFIT3* (Forward primer: GGCTACCTCTATCACCAGATTG; Reverse primer: TCAGCGAGATCGGAGTATGC); *SP140* (Forward primer: TCCGAGACCGCTCCTTCAT; Reverse primer: CAATGCTTCCAGATGTGACCAG); *GADD45B* (Forward primer: CGAGGAGGAGGAGGATGACA; Reverse primer: TCGTGACCAGGAGACAATGC); *MOV10* (Forward primer: CACCATCCTGGACATTCCTAAC; Reverse primer: GTTGCCTTCACGCTCATCTT); *SMARCD3* (Forward primer GAAGAAGACGGCGTGCTATG; Reverse primer ACTGATCTCCTGCTGGTTGG); *JAK2* (Forward primer: ACCTCTAAGTGCTCTGGATTCT; Reverse primer GATCTCGTATGATGGCTCTGAA); *GADD45G* (Forward primer: CTGCTGCGAGAACGACATC; Reverse primer AGGCTGAGCTTCTCCAAGG); *TDRD7* (Forward primer TGGTCTGAGGAGGCTTCTATG; Reverse primer GGTGTCTGGCAACGATGTG); *SETD6* (Forward primer: AGGAACCACTGGAGGAAGAAG; Reverse primer: ATTGGCGTTGTGATTGGCTAA); *PCGF5* (Forward primer: TGACGGAATGCCTCCATACA; Reverse primer: TCAGATTCACGCTCAAGTTCTT); *LMNB1* (Forward primer: CGCTTGGTAGAGGTGGATTCT; Reverse primer: CTGATGACAGTCTGGCATTCTC); *SAP30* (Forward primer: GAGTGATGATGATGGAGGTGAT; Reverse primer: AGTCCTGGTCTGGTTGGTAG); *RSAD1* (Forward primer: CCACCAGTATGAGGTCTCCAA; Reverse primer: GTTGTCAGGCTCCAGTGTCT); DTX3L (Forward primer: GCTGACCTGAACTGTAACCTGCA; Reverse primer: CACCTTCTCAATTCCATCGT); *TLE2* (Forward primer: TTCTTCAGGCTCAATACCACAG; Reverse primer: ATACCGCTCAGACGCTTCA); and Human Endogenous Reference Genes Primers GAPDH (B661104, Sangon Biotech). RNAiso Blood (9113, Takara) was used to obtain total RNA from blood. HiScript III 1 st Strand cDNA Synthesis Kit (R312-01, Vazyme) was used to synthesize the cDNA from total RNA. Taq Pro Universal SYBR qPCR Master Mix (Q712-02, Biosharp) and 7500 Real-Time PCR System (Applied Biosystems) were used to perform the RT-PCR.

### Statistical analyses

Continuous variables are presented as mean ± standard deviation. An independent sample t-test was used to determine the significance of the differences between the two data groups. An analysis of variance was used to determine the significance of the differences between two or more types of data groups. The statistical analysis was performed using the Statistical Package for the Social Sciences software (version 25.0, California, USA). A *p* < 0.05 was considered statistically significant.

## Results

### Clinical profiles in the datasets

The detailed demographic and clinical profiles of the dataset subjects are presented in Tables 1 and 2. The clinical profiles in the GSE83456 dataset included gender and age. In GSE83456 dataset, there were 92 individuals with TB (including 45 with PTB and 47 with EPTB), and 61 healthy control individuals. There was no significant difference in gender or age. The proportion of individuals with PTB was not significantly different from that of individuals with EPTB. In GSE152532 dataset, there were 136 individuals with TB, including 25 active TB (ATB) and 111 latent TB (LTB). The proportion of pre-treatment individuals was not significantly different between ATB and LTB groups.

### Identification of DE-CRs and the correlation between DE-CRs and immune cells

**Fig. 2:**
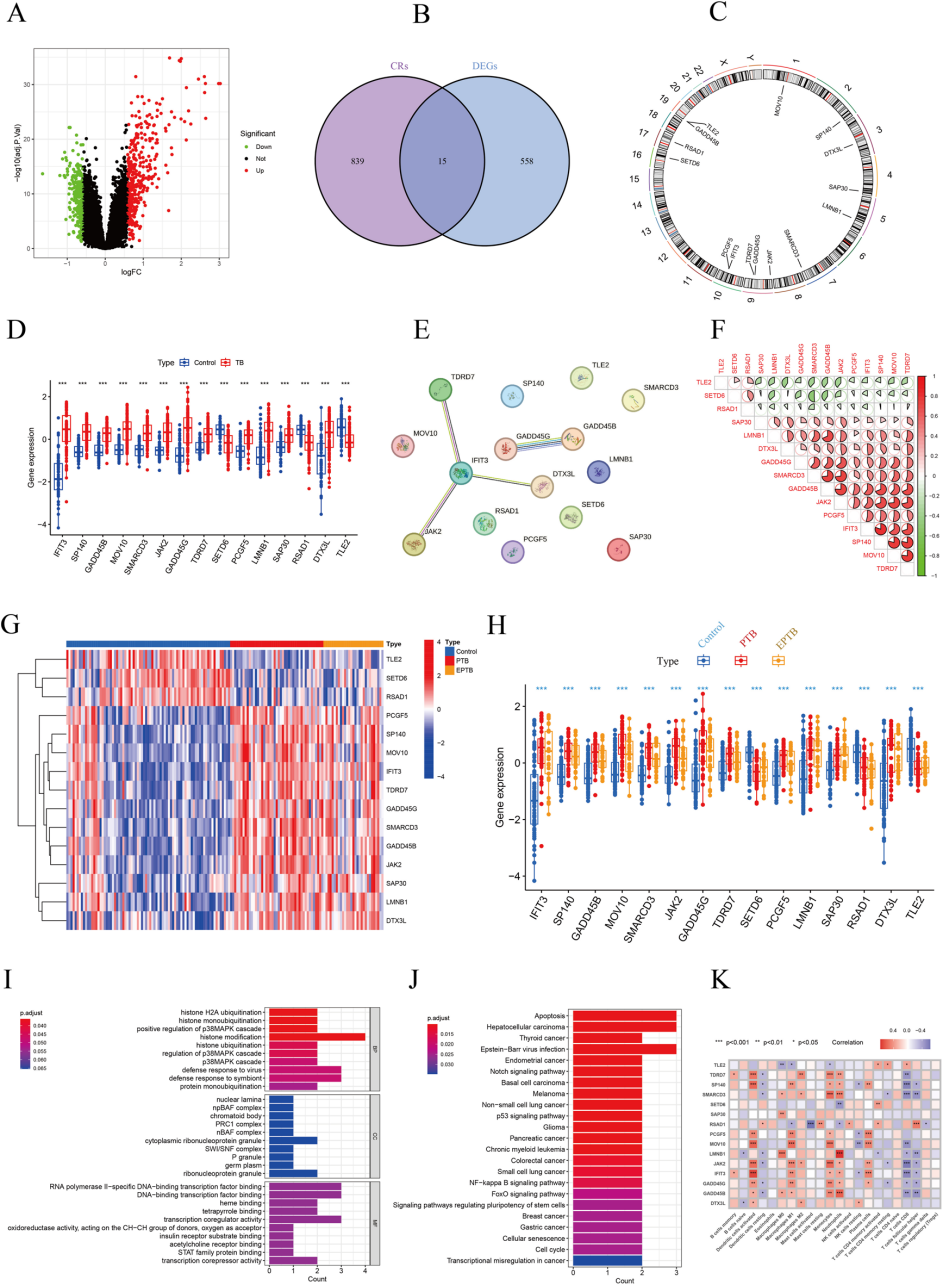
Identification and initial characterization of differentially expressed chromatin regulators (DE-CRs) in TB.**(A)** Volcano plot of differentially expressed genes (DEGs) between patients with TB and healthy controls. Red and blue dots represent significantly up- and down-regulated genes, respectively. **(B)** Venn diagram representing 15 DE-CRs at the intersection of all DEGs and the chromatin regulator gene set. **(C)** Chromosomal distribution of the 15 identified DE-CRs. **(D)** Combined expression profile of the 15 DE-CRs, overall exhibiting significant dysregulation in the TB cohort. (***p < 0.001) **(E)** Protein-protein interaction (PPI) network of DE-CRs, with *IFIT3*, *GADD45B*, and *GADD45G* emerging as core nodes. **(F)** Correlation matrix depicting the co-expression relationships among the 15 DE-CRs, revealing a coordinated regulatory network. **(G-H)** Expression validation of the 15 DE-CRs across healthy controls, pulmonary TB (PTB), and extrapulmonary TB (EPTB) subgroups, confirming their consistent dysregulation in TB irrespective of the disease site. (***,versus control, *p* < 0.001) **(I-J)** Functional enrichment analysis of the DE-CRs. Gene ontology (GO) terms (I) are primarily associated with histone modification and stress-response pathways, while Kyoto encyclopedia of genes and genomes (KEGG) pathways (J) are significantly associated with apoptosis and infection. **(K)** Heatmap of correlations between DE-CRs and infiltrating immune cells, highlighting the significant relationship between epigenetic dysregulation and the immune landscape in TB.

The initial analysis of 574 DEGs identified 239 downregulated and 334 upregulated genes (Fig. 2A). Subsequently, we identified 15 DE-CRs by intersecting DEGs and CRs (Fig. 2B). The positions of these 15 CRs on chromosomes are presented in Fig. 2C. Among the 15 DE-CRs, the expression levels of *IFIT3*, *SP140*, *GADD45B*,* MOV10*, *SMARCD3*, *JAK2*, *GADD45G*, *TDRD7*, *PCGF5*, *LMNB1*, *SAP30*, and *DTX3L* were upregulated in the TB cohort, whereas those of *SETD6*, *RSAD1*, and *TLE2* were downregulated (Fig. 2D). Based on the PPI analysis of these 15 DE-CRs, the core molecules were *IFIT3*, *GADD45B*, and *GADD45G* (Fig. 2E). To determine the interrelationship between these 15 CRs, we calculated the correlation coefficient between genes (Fig. 2F). The strongest correlation was observed with *IFIT3*, which was positively correlated with *SP140* (correlation coefficient = 0.80). Furthermore, positive correlations were observed between *GADD45B* and *JAK2* (correlation coefficient = 0.76), *MOV10* and SP140 (correlation coefficient = 0.79), *MARCD3* and *GADD45B* (correlation coefficient = 0.73), and *TDRD7* and *SP140* (correlation coefficient = 0.78) (Fig. 2F). The expression profile of DE-CRs was further analyzed to identify the DE-CRs within each phenotype (healthy control, PTB, and EPTB). Compared with the control group, the expression of all 15 DE-CRs was significantly altered in PTB and EPTB groups. However, no significant differences were observed between PTB and EPTB groups (Figs. 2G-H). Subsequently, we investigated the biological functions of these 15 CRs. GO analysis revealed that biological functions, including histone H2A ubiquitination, histone monoubiquitination, and positive regulation of the p38 MAPK cascade, were enriched in the TB group (Fig. 2I). KEGG analysis indicated that apoptosis, Notch signaling pathway, and p53 signaling pathway were enriched in the TB group (Fig. 2J). Our previous study observed variations in immune cell infiltration between normal individuals and those with TB^25^. In this study, we identified correlations between immune cells and CRs, including memory B cells, naïve B cells, M0-macrophages, M1-macrophages, M2-macrophages, activated dendritic cells, resting dendritic cells, monocytes, activated mast cells, resting mast cells, neutrophils, plasma cells, activated NK cells, resting NK cells, activated CD4 memory T cells, resting CD4 memory T cells, CD8 T cells, follicular helper T cells, and gamma delta T cells (Fig. 2K).

### CR clusters in TB

**Fig. 3:**
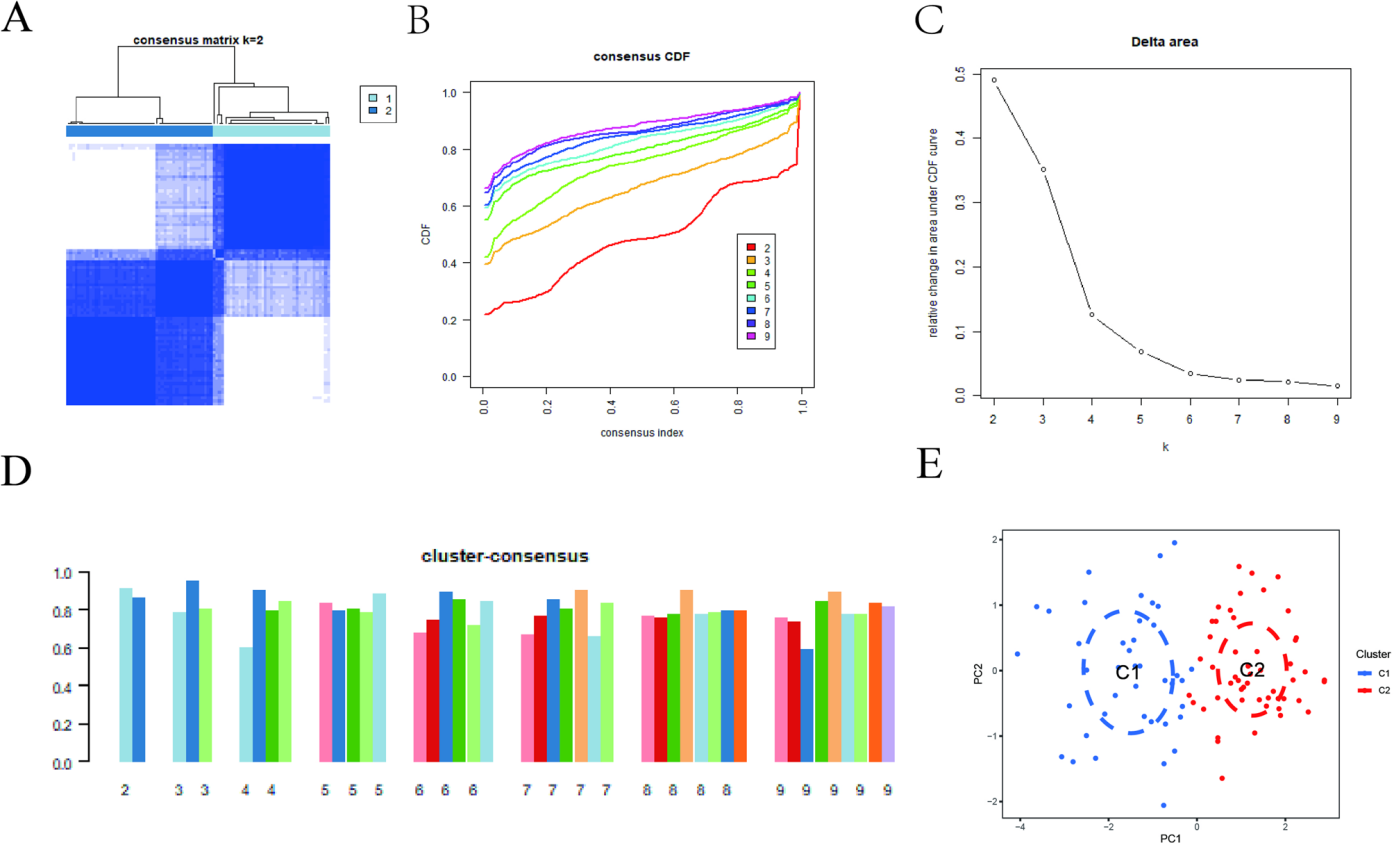
Identification of molecular clusters in TB based on DE-CR expression profiles.**(A)** Consensus matrix heatmap indicating the stability of sample clustering when k = 2, with clearer block-like structures suggesting distinct clusters. **(B)** Cumulative distribution function (CDF) curves for consensus clustering. The minimal change in the CDF curve area between k = 2 and k = 3−9 (panel C) supports k = 2 as the optimal choice. **(C)** Relative change in area under the CDF curve for different k values. A negligible increase after k = 2 indicates that further clustering does not provide significant improvement. **(D)** Cluster-consistency plot indicating that the highest consensus scores for each subtype are achieved at k = 2. **(E)** Principal component analysis (PCA) plot visually confirming the clear separation between the two identified molecular clusters, Cluster 1 (*n* = 41) and Cluster 2 (*n* = 51).

A consensus clustering algorithm was used to categorize 92 TB samples based on the expression patterns of the 15 CRs. Optimal clustering stability was observed when k = 2, as indicated by the consistent pattern of the cumulative distribution function (CDF) curve within a short range of the consensus index, from 0.2 to 0.6 (Figs. 3A-B). Between k = 2 and 9, the area under the CDF curve exhibits the difference between CDF curves for k and k-1 (Fig. 3C). When k = 2, only the optimal consistency score for each subtype was observed (Fig. 3D). Using PCA, the 92 individuals with TB were classified into two distinct cohorts: Cluster 1 (*n* = 41) and Cluster 2 (*n* = 51). Significant differences were observed between these two clusters (Fig. 3E).

### Differential analysis of CRs and immune infiltration between CR clusters

**Fig. 4:**
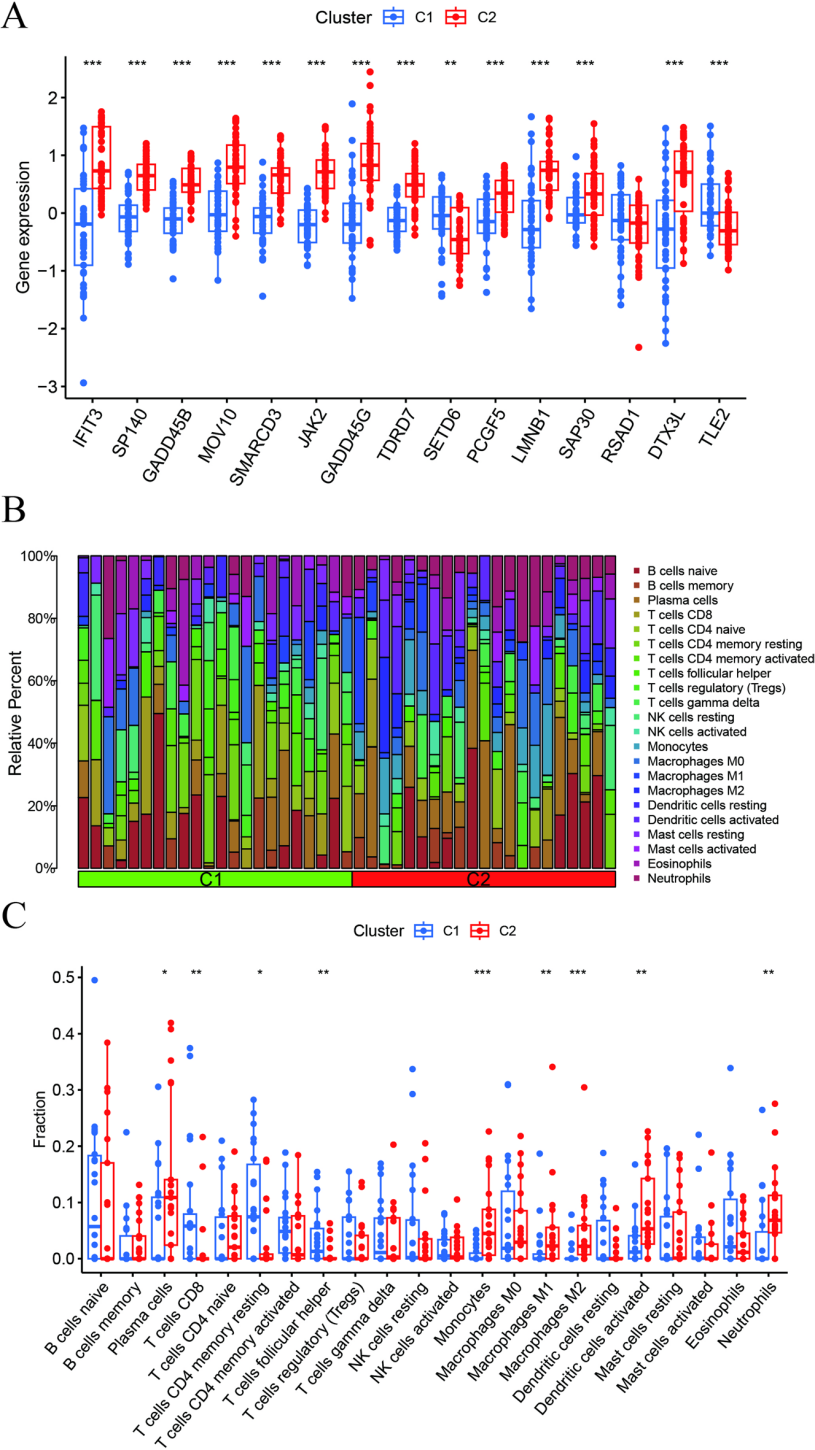
Differential analysis of CR expression and immune infiltration between the two molecular clusters.**(A)** Expression levels of the 15 DE-CRs in Clusters 1 and 2. Cluster 2 exhibits significantly upregulated expression of most pro-inflammatory/stress-related CRs (*IFIT3* and *GADD45B/G*), while *SETD6* and *TLE2* are downregulated. **(B)** Heatmap displaying the relative abundance of 22 immune cell types in the two clusters, exhibiting distinct immune microenvironment landscapes. **(C)** Box plots comparing the infiltration levels of key immune cell populations between clusters. Cluster 2 is enriched for innate immune cells (monocytes, macrophages, and neutrophils), whereas Cluster 1 exhibits higher levels of T cell subsets (CD8 + T cells and resting memory CD4 + T cells).

To investigate the variations in molecular characteristics between the clusters, a comprehensive assessment of Clusters 1 and 2, including the 15 CRs, was conducted. The distinct CR expression landscapes of Clusters 1 and 2 are depicted in Fig. 4A. Cluster 2 exhibited significantly upregulated expression of *IFIT3*, *SP140*, *GADD45B*, *MOV10*, *SMARCD3*, *JAK2*, *GADD45G*, *TDRD7*, *PCGF5*, *LMNB1*, *SAP30*, and *DTX3L;* however, significantly downregulated *SETD6* and *TLE2* expression (Fig. 4A). Additionally, immune cell infiltration analysis identified unique variations in the immune microenvironments between Clusters 1 and 2 (Fig. 4B). Plasma cells, monocytes, M1-macrophages, M2-macrophages, activated dendritic cells, and neutrophils exhibited increased abundance in Cluster 2, whereas CD8 + T and resting memory CD4 + T cells demonstrated reduced abundance (Fig. 4C).

### Biological functions and pathway activities

**Fig. 5:**
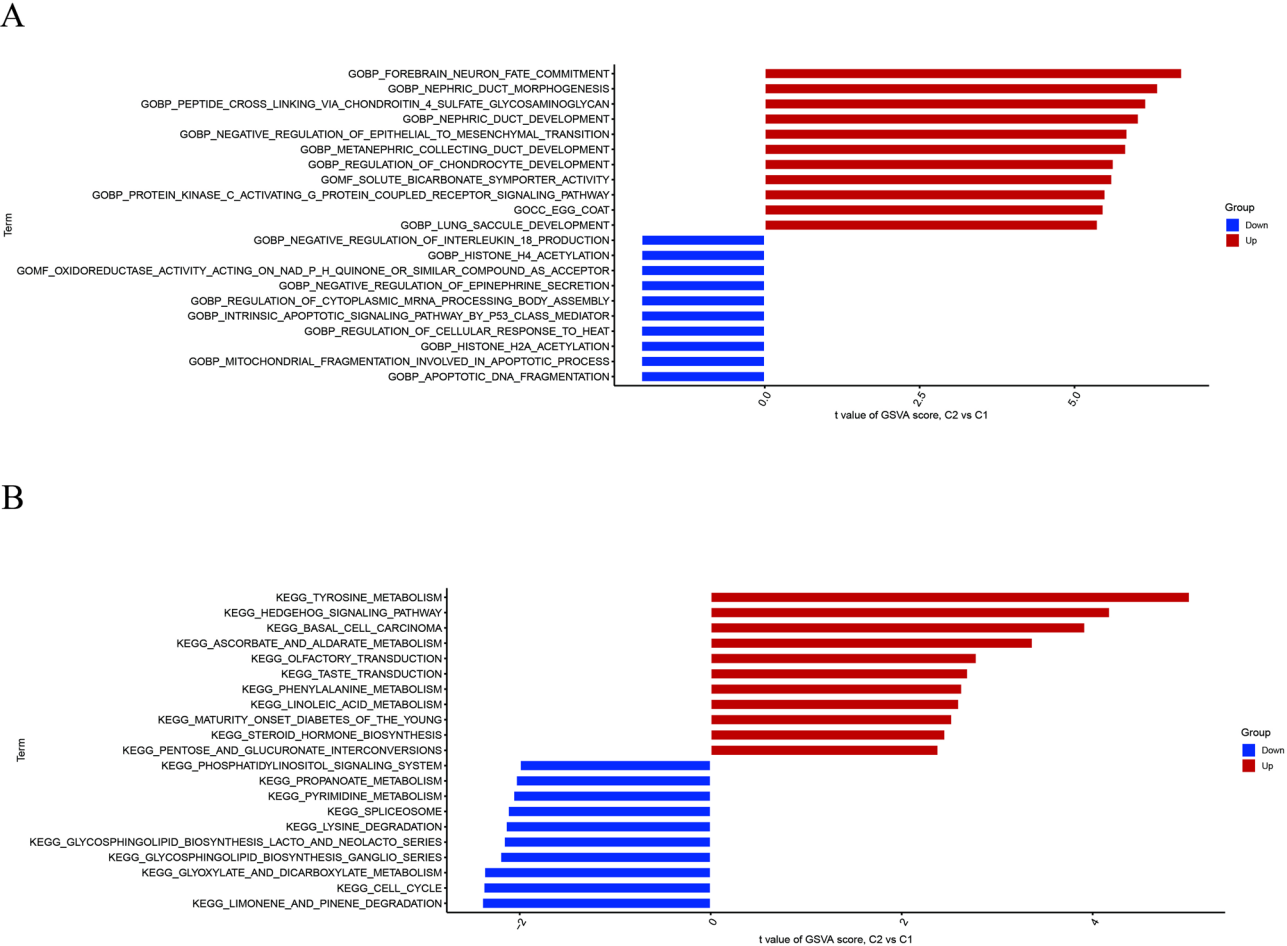
Enriched biological functions and pathways in CR-based molecular clusters.**(A)** Gene Set Variation Analysis (GSVA) reveals significant differences in biological processes between clusters. Cluster 2 is enriched in developmental and metabolic processes, while Cluster 1 depicts downregulation of immune and inflammatory responses (interleukin-18 synthesis). **(B)** GSVA of KEGG pathways highlighting divergent pathway activities. Cluster 2 exhibits activation of metabolic and signaling pathways (hedgehog signaling), whereas Cluster 1 is characterized by active cell cycle and degradation pathways.

The t-value obtained from GSVA was used to evaluate and compare the differences in biological activities between Clusters 1 and 2. Cluster 2 was active in forebrain neuron fate determination, nephric duct morphogenesis, peptide cross-linking through chondroitin 4 sulfate glycosaminoglycan, and nephric duct development. Cluster 1 exhibited downregulated interleukin-18 synthesis, histone H4 acetylation, negative regulation of epinephrine secretion, and oxidoreductase activity with NADPH quinone or a similar chemical as an acceptor (Fig. 5A). Pathway activity results suggested that tyrosine metabolism, the hedgehog signaling pathway, basal cell carcinoma, and ascorbate and aldarate metabolism were active in Cluster 2. In contrast, limonene and pinene degradation, cell cycle, glyoxylate, and dicarboxylate metabolism were active in Cluster 1 (Fig. 5B).

### Gene module screening and co-expression network construction

**Fig. 6:**
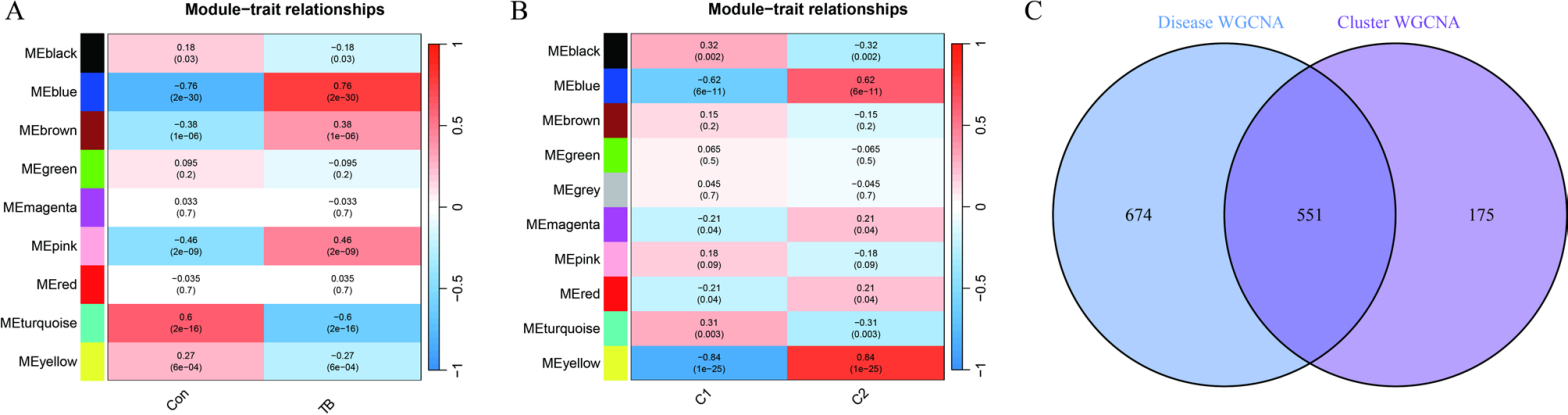
Weighted gene co-expression network analysis (WGCNA) identifies key modules associated with TB and CR clusters.**(A)** Module-trait relationships between co-expression modules and clinical status (Control versus TB). The blue module presents the highest positive correlation with TB. **(B)** Module-trait relationships between co-expression modules and CR clusters. The yellow module is most significantly associated with the cluster phenotype. **(C)** Venn diagram identifying 551 overlapping genes between TB-related blue module and cluster-related yellow module, representing a core gene set potentially driving both disease status and molecular heterogeneity.

WGCNA algorithm was used to generate co-expression networks and modules for healthy individuals and individuals with TB, aiming to identify crucial gene modules associated with TB. Gene expression variation was computed in dataset GSE83456, and the subset of genes with the highest variance, specifically the top 25%, was selected for subsequent analysis. The dynamic cutting algorithm exhibited 10 different color co-representation modules, and the TOM heatmap was generated. Of the identified genes, the blue module comprised 1,225 genes that exhibited the highest level of correlation with TB (Fig. 6A). Furthermore, WGCNA was used to analyze significant gene modules with a close relationship with DE-CR clustering. The correlation analysis of modular clinical features (Clusters 1 and 2) revealed that 726 genes in the yellow module were highly correlated with TB clusters (Fig. 6B). Finally, 551 overlapping genes were identified between the two modules using “Venn” R package (Fig. 6C).

### Construction of ML models

**Fig. 7:**
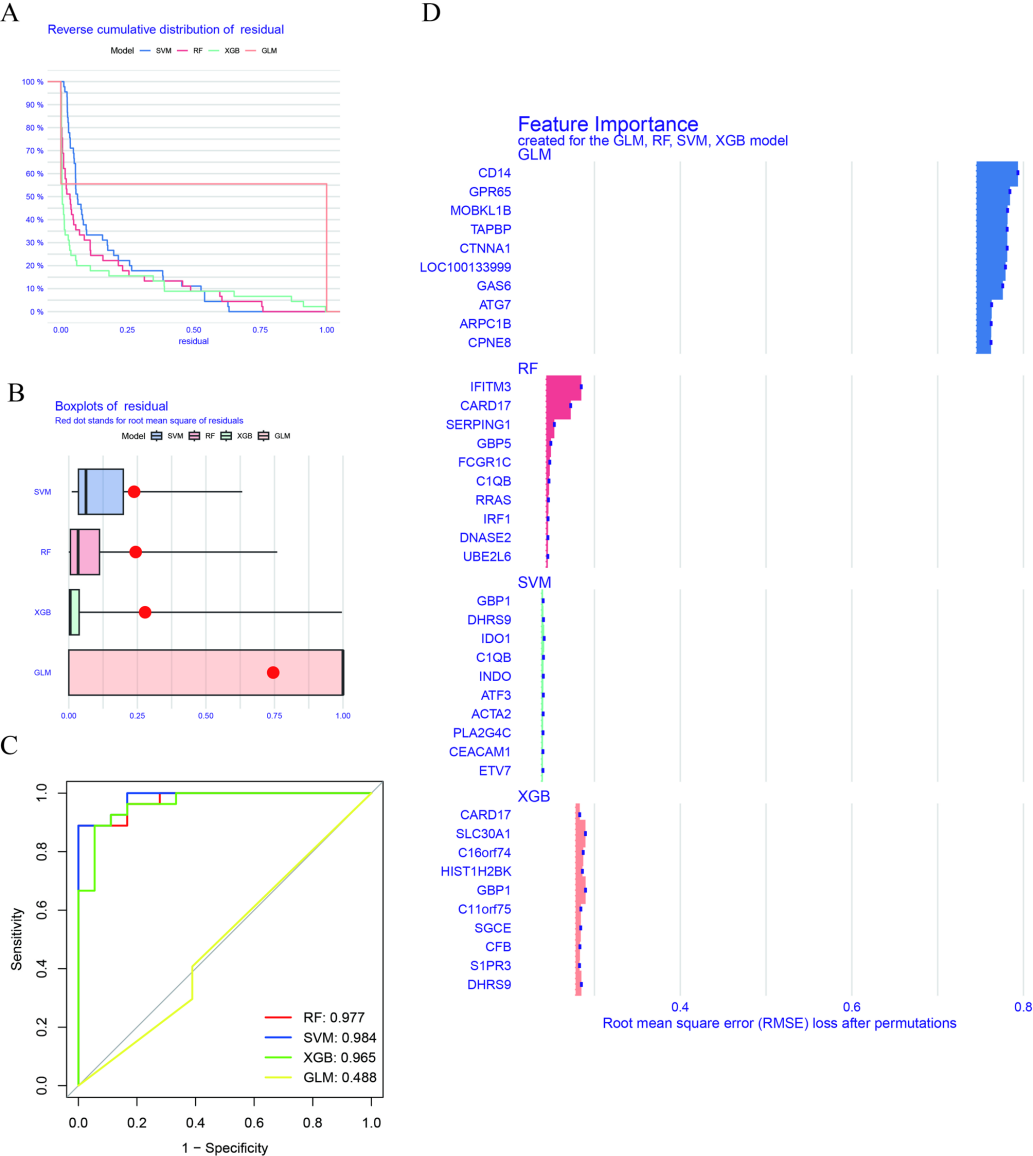
Construction and evaluation of machine learning models for distinguishing TB clusters.**(A-B)** Residual distribution plots for the four ML models. The XGBoost (XGB) and Support Vector Machine (SVM) models exhibit the smallest and most centered residuals, indicating better fit and prediction accuracy. **(C)** Variable importance plot displaying the top 15 predictive genes for each model, ranked by their contribution to the model’s performance. **(D)** Receiver operating characteristic (ROC) curves of the four models using 5-fold cross-validation. Random Forest (RF), SVM, and XGB models achieve excellent performance (AUC > 0.96), significantly outperforming the Generalized Linear Model (GLM).

We constructed four ML models (RF, SVM, GLM, and XGB) using genes ranked by differential expression between the two TB clusters. XGB and RF models exhibited the lowest residuals (Figs. 7A-B). Evaluation based on the area under the receiver operating characteristic curve (AUC) revealed that RF, SVM, and XGB models performed excellently, with AUC values of 0.977, 0.984, and 0.965, respectively. In contrast, the GLM model performed poorly (AUC = 0.488) (Fig. 7C). This superior performance was consistent under 5-fold cross-validation (Fig. 7D). This indicates that the XGB model is less susceptible to overfitting and more accurately represents unseen data, which is a critical criterion for a clinically applicable model. Based on residuals and AUC, the XGB ML model demonstrated a superior ability to differentiate between individuals with TB, exhibiting distinct characteristics. Finally, the five most significant genes (*DHRS9*, *HIST1H2BK*, *C16orf74*, *SLC30A1*, and *GBP1*) were extracted from the genome.

### Construction of a nomogram model

**Fig. 8:**
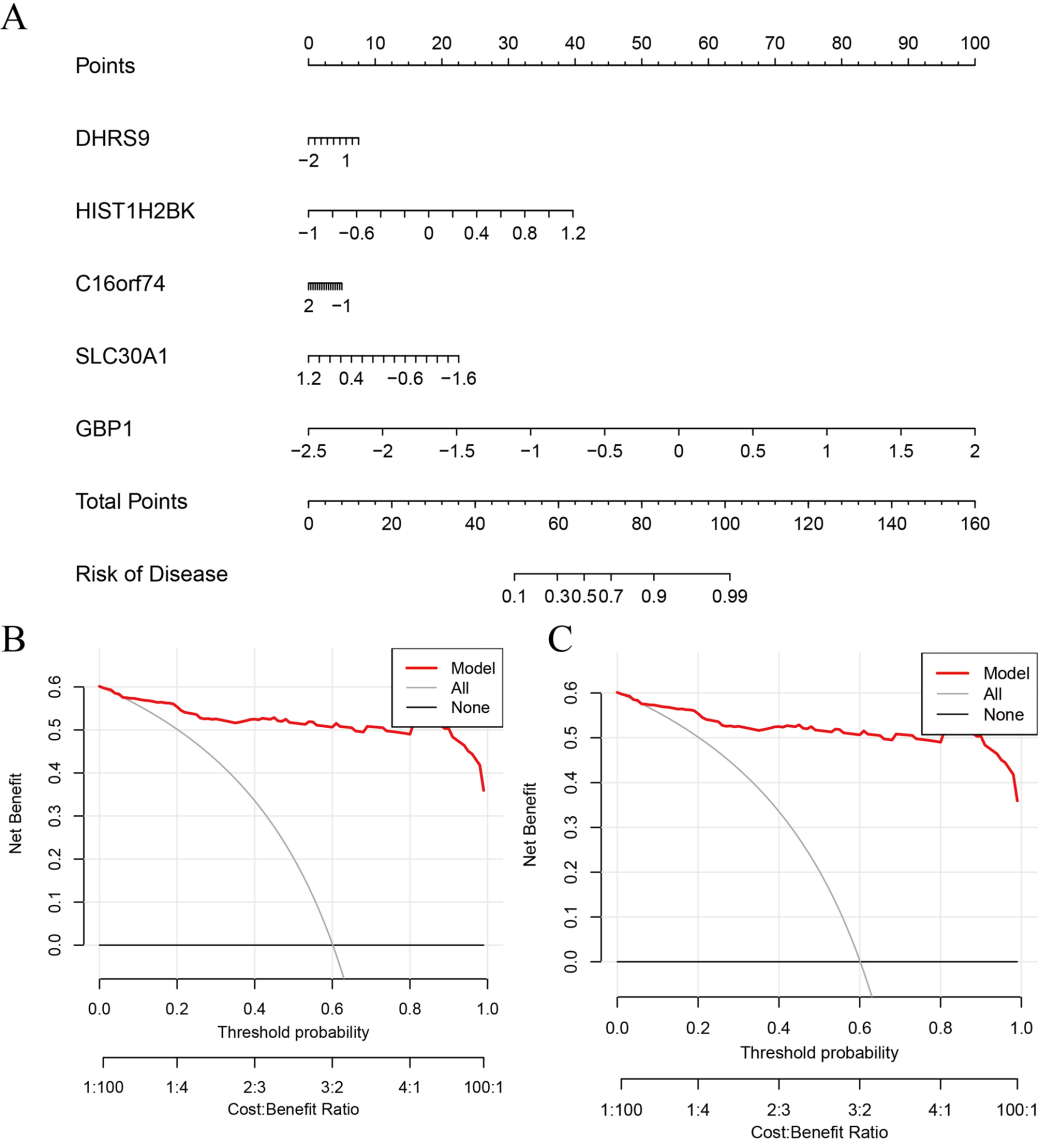
Clinical translation potential of the XGBoost model.**(A)** A nomogram developed to predict an individual’s probability of belonging to a specific TB molecular cluster (Cluster 1 or 2) based on the expression levels of the five key genes (*DHRS9*,* HIST1H2BK*,* C16orf74*,* SLC30A1*,* and GBP1*) from the XGB model. **(B)** Calibration curve of the nomogram. The close fit of the solid line (observed outcomes) to the dashed line (ideal prediction) indicates excellent agreement between predicted and actual probabilities. **(C)** Decision curve analysis (DCA) for the nomogram. The red line represents that using the nomogram for clinical decision-making provides a higher net benefit across a wide range of threshold probabilities compared to the “treat all” or “treat none” strategies (gray lines).

The nomogram model was built to determine the risk of CR-cluster TB cases in a cohort of 92 individuals (Fig. 8A). The effectiveness of the nomogram model prediction was evaluated using the calibration curve and DCA. The calibration curve illustrates that the actual risk of the TB cluster is less than the predicted risk (Fig. 8B). DCA indicates that the nomogram exhibits a significant degree of accuracy, making it a valuable tool for guiding clinical decision-making (Fig. 8C).

### Assessment of the ML models

**Fig. 9:**
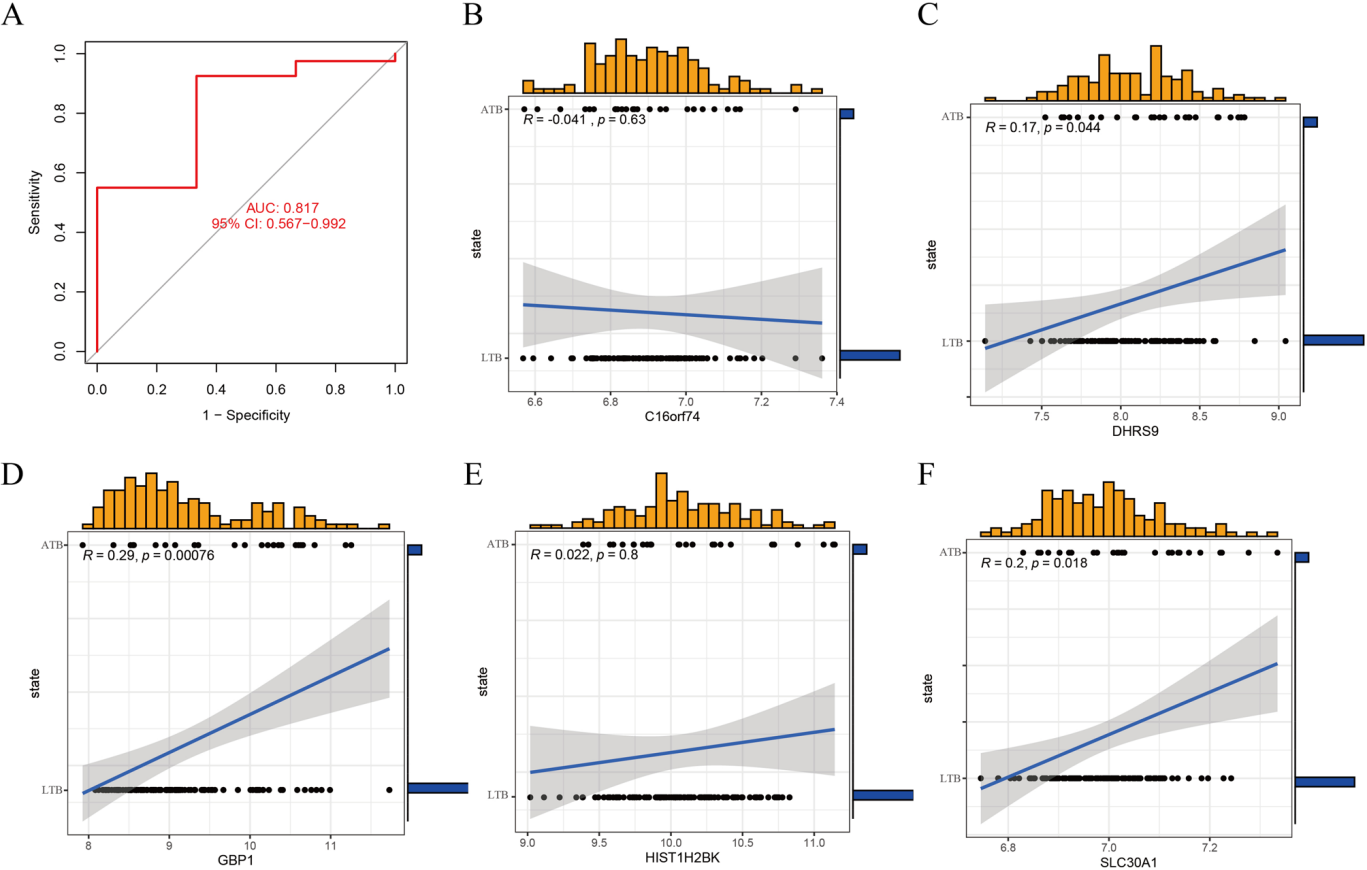
Validation of the five-gene signature for distinguishing active and latent TB.**(A)** Receiver operating characteristic (ROC) curve evaluating the diagnostic performance of the five-gene XGB signature in the external validation dataset (GSE152532), achieving an AUC of 0.817. **(B−F)** Correlation analysis between the expression of each of the five genes and the clinical status of active TB (ATB) versus latent TB (LTB). The expression of *DHRS9* (B), *SLC30A1* (E), and *GBP1* (F) exhibits a significantly positive correlation with ATB, highlighting their potential as biomarkers for disease activity.

The XGB model was chosen as the final model for further analysis, despite the SVM model achieving a marginally higher AUC (0.984) on the internal training set than the XGB model (0.965). This decision was primarily based on its superior generalizability and more robust performance when validated on the external dataset GSE152532. On this independent validation set, the XGB model exhibited a higher predictive power (AUC = 0.817, Fig. 9A) compared to the SVM model (AUC = 0.608, Fig. 1S). The ML model accuracy was validated using GSE152532 dataset. Five genes from the XGB model (*DHRS9*, *HIST1H2BK*, *C16orf74*, *SLC30A1*, and *GBP1*) performed well in the ROC curve of the prediction model, with an AUC value of 0.817 (Fig. 9A). TB cases in GSE152532 dataset were classified as LTB or ATB. Using these five genes, LTB and ATB were predicted based on clinical characteristics (Figs. 9B–F). Furthermore, *DHRS9* (*R* = 0.17), *SLC30A1* (*R* = 0.20), and *GBP1* (*R* = 0.29) exhibited positive correlations with ATB.

### Identification of DE-CRs in individuals with TB

**Fig. 10:**
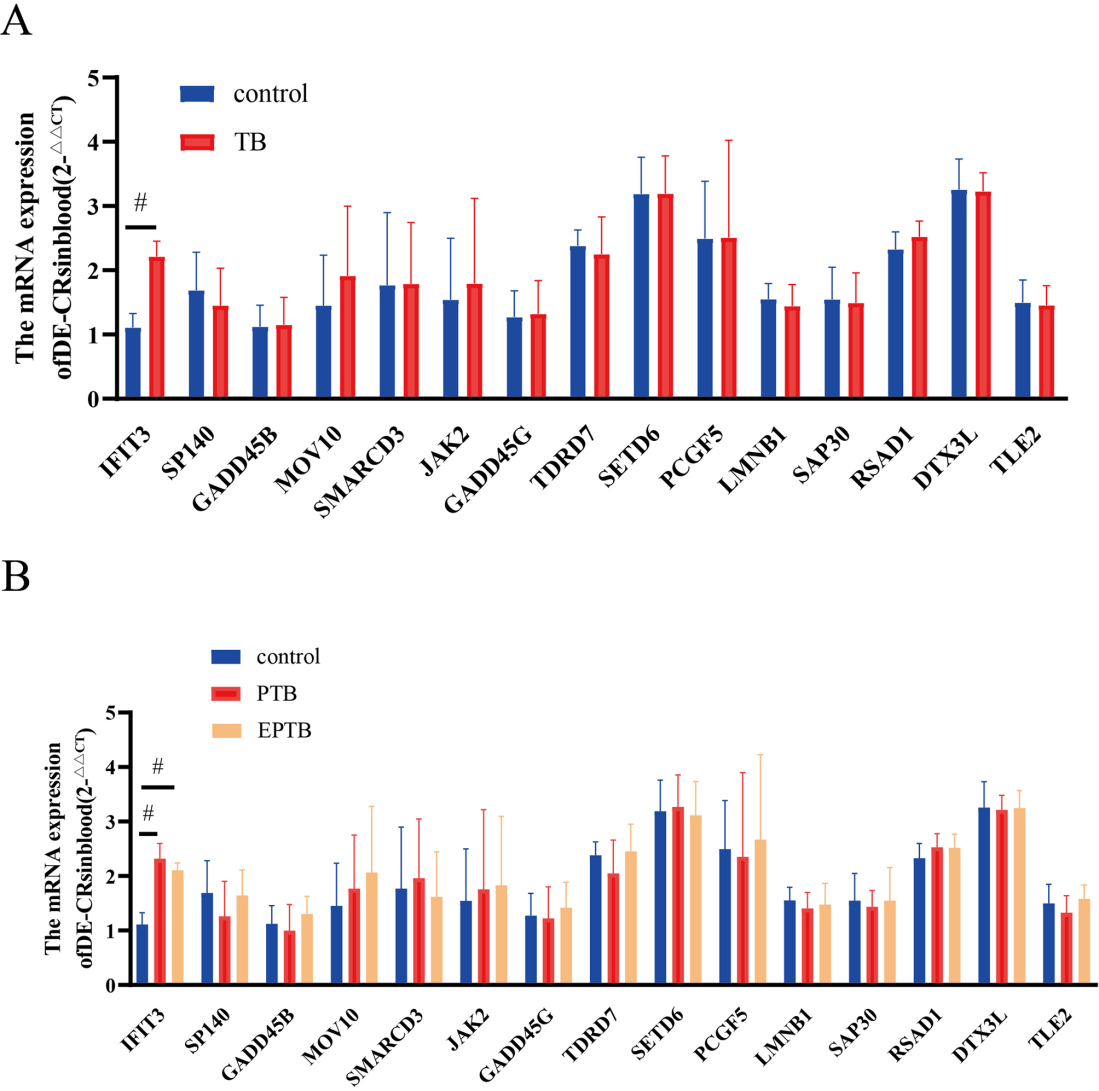
Clinical validation of DE-CRs in patient blood samples.**(A-B)** Reverse transcription-quantitative PCR (RT-qPCR) validation of the 15 DE-CRs in blood samples from an independent clinical cohort. **(A)** Comparison between healthy controls (*n* = 15) and the overall TB group (*n* = 18, combining PTB and TBM). Only *IFIT3* exhibits significant upregulation in the TB group, confirming its role as a promising blood-based biomarker. (versus control group, #< 0.01) **(B)** Comparison across healthy controls (*n* = 15), pulmonary TB (PTB, *n* = 9), and tuberculous meningitis (TBM, *n* = 9) subgroups. *IFIT3* is significantly upregulated in PTB and TBM patients compared to controls. However, it does not differ between the two clinical forms of TB, supporting its utility as a pan-TB biomarker (versus control group, #< 0.01).

The clinical characteristics of the individuals are presented in Table 3. *IFIT3* expression was significantly upregulated in individuals with TB (including PTB and TBM) compared to healthy controls (*p* < 0.05; Fig. 10A). However, no significant differences were observed in the expression of the other 14 genes compared to the control group (*p* > 0.05; Fig. 10A). Compared with the control group, there were significant differences in *IFIT3* in the PTB and EPTB groups; however, there was no significant difference between the PTB and EPTB groups (versus control group; *p* ˂ 0.05; Fig. 10B). Notably, this clinical validation was performed on a relatively small cohort (*n* = 18). While the results for *IFIT3* are statistically significant and consistent with our bioinformatics findings, the limited sample size restricts the generalizability of these conclusions. It underscores the preliminary nature of this validation. Future studies with larger cohorts are required to confirm these observations.

## Discussion

Previous studies have reported that epigenetic changes are essential in *M.tb.-*host interactions. These changes can affect the physiological processes of *M.tb*. and the immune regulatory mechanisms of the host^13–15^. Consequently, providing a novel perspective for the clinical diagnosis and treatment of TB requires investigation of the potential mechanism of upstream CRs in epigenetics and identification of CRs with diagnostic significance.

### CRs and TB

We aimed to identify TB-associated CRs as potential novel biomarkers, given the suboptimal diagnostic accuracy for PTB and the even greater challenges in diagnosing EPTB^6–8^. Using GSE83456 dataset, we identified 15 DE-CRs. Notably, the expression levels of *IFIT3*, *SP140*, *GADD45B*, *MOV10*, *SMARCD3*, *JAK2*, *GADD45G*, *TDRD7*, *PCGF5*, *LMNB1*, *SAP30*, and *DTX3L* were upregulated in the TB cohort, while *SETD6*, *RSAD1*, and *TLE2* were downregulated.

Among these, *IFIT3*, *GADD45B*, and *GADD45G* were identified as core molecules in the PPI network, suggesting their central role in the CR regulatory landscape of TB. This finding is mechanistically significant. *IFIT3* is a well-established interferon-stimulated gene whose elevation in TB^34,35^ can drive *M.tb.-*induced macrophage death through type I interferon signaling^36,37^. Our analysis confirms a close relationship between *IFIT3* and macrophage infiltration, supporting the hypothesis that *IFIT3*-mediated macrophage death contributes to TB immunopathology. The concurrent upregulation of *GADD45B* and *GADD45G* suggests an elevated level of cellular stress. *GADD45B* is known to activate the ROS-p38MAPK cascade, a pathway associated with cellular damage and apoptosis^38,39^. Additionally, *GADD45G* is involved in DNA demethylation in response to stress^40^. Therefore, it is hypothesized that these factors contribute to the oxidative stress and mitochondrial dysfunction observed in *M.tb.-*infected cells, potentially contributing with *IFIT3* to determine the fate of macrophages, a critical determinant of TB progression^41,42^.

Furthermore, our correlation analysis exhibited a coordinated network among these DE-CRs. The strong positive correlation between *IFIT3* and *SP140* (a transcriptional regulator associated with immune dysregulation and *M.tb.* susceptibility^43,44^ suggests a synergistic disruption of the host transcriptional response. Similarly, the association between *GADD45B* and *JAK2* is particularly intriguing. *JAK2* mediates cytokine signaling and *M.tb.-*induced macrophage apoptosis^45^, suggesting a potential convergence of cellular stress (*GADD45B*) and cytokine signaling (*JAK2*) pathways in driving immune cell death. The role of *SMARCD3*, which correlates with *GADD45B* and has been proposed as a TB marker^46^, requires further investigation in this context. Our functional enrichment analysis strongly supports this mechanistic model. GO analysis indicated that these DE-CRs are primarily involved in the p38MAPK cascade (*GADD45B*), defense response to symbionts (*IFIT3*), and histone modification (*JAK2* and *DTX3L*), the latter being a key promoter of monocyte function in TB^47^. KEGG analysis further established a correlation between these CRs, apoptosis, and Epstein-Barr virus infection pathways, which have been previously associated with TB pathogenesis^41,42,48,49^. The association with hepatocellular carcinoma, while more speculative, is supported by cases of hepatic TB complicating hepatocellular carcinoma^50^ and the known role of CRs in cancer pathogenesis^51^.

Finally, and crucially for their diagnostic potential, we found that the expression profiles of these 15 DE-CRs did not differ significantly between PTB and EPTB subtypes. This consistency, in conjunction with the absence of significant demographic confounders in our dataset, highlights their potential utility as pan-TB biomarkers, capable of identifying TB infection irrespective of its primary site.

### The relationship between CRs and immune cells in TB

Host resistance to *M.tb.* infection requires the coordinated activity of multiple immune cell subsets. Our findings suggest that CRs are essential in orchestrating this response. It is well established that perturbations in T cell, B cell, monocyte, and dendritic cell subsets can influence TB prognosis and facilitate the transition from latent to active disease^52^; however, the upstream regulatory mechanisms are still being investigated. In this study, we move beyond this general concept by providing direct evidence that the expression of specific DE-CRs is significantly correlated with alterations in immune cell infiltration. Our analysis revealed that the DE-CR signature we identified is closely associated with the abundance of key innate immune players, including macrophages (M0, M1, and M2), neutrophils, and dendritic cells. This is highly relevant, as the balance between macrophage polarization states (pro-inflammatory M1 versus immunoregulatory M2) is crucial for containing *M.tb.*^53^. Additionally, neutrophils exhibit a dual role, both controlling infection and contributing to tissue damage^54^.

We hypothesize that dysregulated CRs are not merely correlates; rather, they can actively shape the immune landscape in TB. The significant correlation between *IFIT3* and macrophage abundance suggests that this interferon-induced CR could affect macrophage function or fate, potentially through mechanisms, including regulating genes involved in cell death or inflammation. Furthermore, the correlation between diverse lymphocyte and myeloid populations and other DE-CRs (JAK2 and SP140) suggests that epigenetic modulation plays a more extensive role in determining the identity, recruitment, and functional capacity of immune cells during infection.

The known functions of these CRs support this perspective. *SP140* is a nuclear protein essential for macrophage transcriptional responses to infection^43,44^. *JAK2* is a master regulator of cytokine signaling that controls immune cell activation and differentiation^47^. Consequently, the CR-mediated modulation of histone marks and DNA accessibility could be a key mechanism through which *M.tb.* indirectly manipulates the host immune environment to facilitate its survival. In conclusion, our findings contribute to the existing body of knowledge regarding the role of immune cells in tuberculosis. We propose that a network of dysregulated CRs contributes to pathogenesis by epigenetically reprogramming the immune microenvironment, thereby affecting the functionality of macrophages, neutrophils, and other critical cells involved in the anti-mycobacterial response.

### Molecular clusters in TB

Molecular clustering has emerged as a robust tool for delineating disease heterogeneity^55^. Based on their DE-CR expression profiles, we divided individuals with TB into two distinct molecular clusters. This stratification was biologically imperative, as demonstrated by significant differences in their enriched pathways and immune microenvironments. Cluster 2 exhibited activation of pathways associated with peptide cross-linking and nephric duct development, suggesting a potential involvement of the nervous and urinary systems. Conversely, Cluster 1 exhibited enrichment in the negative regulation of interleukin-18 production and histone H4 acetylation, indicating a more severe immunosuppressive or dysregulated inflammatory state. Furthermore, pathway analysis revealed activated tyrosine metabolism and the hedgehog signaling pathway in Cluster 2. The latter finding is particularly relevant, as an upregulated hedgehog pathway has been demonstrated to protect the blood-brain barrier in TB^56^, suggesting that individuals in Cluster 2 could possess a mechanistic defense against tuberculous meningitis. In contrast, Cluster 1 was characterized by the active degradation of limonene and pinene, as well as cell cycle pathways, the implications of which require further investigation.

### Significance of ML models in TB

TB heterogeneity requires models that incorporate multifaceted data for accurate prediction. Our multifactor ML approach, which integrates the complex relationships between variables, provides increased reliability over single-parameter models^57,58^. Previous studies have successfully implemented ML using meteorological or radiomic features to predict PTB^59,60^; however, their focus was limited. Additionally, their models are not directly applicable to all TB forms or to the molecular subtyping we are currently pursuing here. The optimal ML algorithm is highly context-dependent, varying with the gene set used and patient demographics^61–63^. Therefore, we systematically evaluated four classifiers (RF, SVM, GLM, and XGB) using our CR-based gene expression profiles. Evaluation based on the AUC revealed that RF, SVM, and XGB models performed excellently, with AUC values of 0.977, 0.984, and 0.965, respectively.

The SVM model achieved a marginally higher (AUC = 0.984) than the XGB model (AUC + 0.965) on the training set; however, the XGB model was selected as the final model based on the following considerations: First, the XGB model exhibited a superior and more stable residual distribution compared to other models, indicating a more robust and reliable fit to the data. Second, and most importantly, the XGB framework provides intrinsic and straightforward feature importance rankings. This capability was essential for our study, as it enabled us to identify and prioritize the most significant predictor genes responsible for the accurate differentiation of TB clusters. In contrast, SVM models are inherently less interpretable and do not provide a direct and intuitive measure of feature importance, despite their power. This is a significant disadvantage for biomarker discovery studies where identifying key drivers is a primary objective.

Consequently, given its robust performance, stability, and superior interpretability, the XGB model was considered the most suitable tool for achieving our research goals. Subsequently, five genes (*DHRS9*, *HIST1H2BK*, *C16orf74*, *SLC30A1*, and *GBP1*) were selected as the most significant predictors for the final XGB model.

### Significant variables of ML in differentiating ATB/LTB

Differentiating between ATB and LTB is critical for clinical management; however, the current tests (TST and IGRA) cannot evaluate infectivity or treatment response, which presents a challenge^64–69^. To address this, we used GSE152532 dataset, which includes ATB and LTB samples with treatment data. Using the five-gene signature from our XGB model, we observed that *DHRS9*, *SLC30A1*, and *GBP1* expression levels exhibited significantly positive correlation with ATB status. This aligns with their biological roles: *DHRS9* generates all-trans retinoic acid, a molecule known to modulate macrophage response to *M.tb.*^70,71^, and *GBP1* is implicated in host defense against microbial infections^72^. Thus, these genes represent promising candidates for developing biomarkers that can not only differentiate ATB from LTB but also potentially monitor treatment efficacy.

### Diagnostic value of CRs for TB

Improving the diagnostic accuracy for PTB and EPTB remains a paramount challenge. Our clinical validation focused on the top candidate from our bioinformatics analysis, *IFIT3*. We confirmed that *IFIT3* expression was significantly upregulated in the blood of individuals with TB (including PTB and EPTB subgroups) compared to healthy controls, highlighting its potential as a general TB biomarker. Notably, *IFIT3* expression did not differ significantly between PTB and EPTB (TBM) individuals. This indicates that, although *IFIT3* is a valuable marker for differentiating healthy individuals from those with any form of TB, it cannot differentiate between TB manifestations. The discrepancy between the clear bioinformatics signature and the more focused clinical validation result is likely attributed, at least in part, to the limited statistical power of our small clinical cohort (*n* = 18). The small sample size, which precludes a robust assessment of its ability to differentiate between TB subtypes, increases the risk of overestimating the effect size, despite the successful validation of IFIT3 elevation in patients with TB. Therefore, these clinical findings should be interpreted as preliminary but promising, requiring confirmation in larger, multi-center studies with greater statistical power.

### Potential for clinical translation of ***IFIT3***

The consistent upregulation of *IFIT3* in patients with TB highlights its potential for clinical development. An immediate application could be a blood-based qPCR assay, providing an objective and quantifiable method to supplement existing diagnostics, including smear microscopy or interferon gamma release assay (IGRA), especially in challenging cases. Developing a rapid, point-of-care test format could be the focus of future research to achieve a more extensive impact in resource-limited settings. The potential of IFIT3 as a pan-TB biomarker to enhance the diagnosis of extrapulmonary TB is particularly promising, as current methods are frequently insufficient. Translating this finding into a validated diagnostic tool requires further development and large-scale clinical validation.

### Prospects and limitations

Based on this study, certain findings hold potential for clinical translation. The five-gene signature (*DHRS9*,* HIST1H2BK*,* C16orf74*,* SLC30A1*, and *GBP1*) derived from our XGBoost model presents a promising foundation for developing a novel qPCR assay. This assay could potentially be integrated into the current diagnostic workflow to supplement existing tests (including TST and IGRA) for more accurate differentiation between ATB and LTB, and potentially for monitoring treatment response. First, *IFIT3* expression in blood could serve as a diagnostic biomarker for TB, including both PTB and TBM. Second, the XGBoost model, built on a five-gene signature (*DHRS9*, *HIST1H2BK*, *C16orf74*, *SLC30A1*, and *GBP1*), exhibits promise for predicting TB risk and subtypes. Specifically, *DHRS9*, *SLC30A1*, and *GBP1* can be useful in differentiating ATB from LTB.

However, this study has certain limitations that must be acknowledged. The most significant is the small sample size (*n* = 18) for our clinical validation, which limits the statistical power of our conclusions regarding the diagnostic utility of individual CRs, including *IFIT3*. Furthermore, although no significant differences in gender and age were observed between groups in the available dataset, the lack of individual-level covariate data prevented us from performing a formal multivariate analysis to completely rule out potential confounding effects of these demographic factors. Additionally, we used GSE152532 dataset for external validation of our ML model; identifying molecular clusters was primarily based on GSE83456 dataset. Future studies incorporating additional datasets with CR expression profiles would be valuable for further validating and refining these clusters. Fourth, the correlation analyses between DE-CRs and immune cells were performed without adjustment for multiple testing. In this exploratory analysis, this method enabled the identification of potentially intriguing relationships; however, it also increased the likelihood of Type I errors. These correlation findings should therefore be interpreted as preliminary and require validation in independent cohorts. Therefore, future studies with larger, multi-center cohorts and detailed clinical metadata are required to validate these biomarkers and models and to comprehensively determine their clinical diagnostic significance.

## Conclusion

In conclusion, our study demonstrated a correlation between CRs and infiltrating immune cells, highlighting significant heterogeneity in immunity among individuals with TB across distinct CR clusters. Based on five genes (*DHRS9*, *HIST1H2BK*, *C16orf74*, *SLC30A1*, and *GBP1*), the XGB model was selected as the optimal ML model for accurately determining TB subtype and disease status (active or latent). Our study established the role of CRs in TB and contributed to a deeper understanding of the molecular mechanisms underlying the heterogeneity observed in TB.

**Table 1:** Detailed demographic and clinical profiles of GSE83456 dataset.

		*N*	Male (*N*, %)	Female (*N*, %)	Age(years, mean ± SD)
Control		61	33 (54.10)	28(45.90)	35.80 ± 11.60
TB	PTB	45	26(57.78)	19(42.22)	38.87 ± 15.22
	EPTB	47	29(61.70)	18(38.30)	36.30 ± 11.31

**Table 2:** Detailed demographic and clinical profiles of GSE152532 dataset.

		*N*	Pre-treatment (*N*, %)	Post-treatment (*N*, %)
Control		11	/	/
TB	ATB	25	17(68.00)	8(32.00)
	LTB	111	69(62.16)	42(37.84)

**Table 3:** Clinical characteristics of the individuals.

		*N*	Male (*N*, %)	Female (*N*, %)	Age(years, mean ± SD)
Control		15	12(80)	3(20)	47.07 ± 9.24
TB	PTB	9	7(77.78)	2(22.22)	44.22 ± 7.74
	EPTB	9	7(77.78)	2(22.22)	45.22 ± 10.37

## Supplementary Information

Below is the link to the electronic supplementary material.

Supplementary Material 1

Supplementary Material 2

## Data Availability

Two datasets (GSE83456 and GSE152532) used in this study were downloaded from GEO (GSE83456 dataset: https://www.ncbi.nlm.nih.gov/geo/query/acc.cgi? acc=GSE83456; GSE152532: https://www.ncbi.nlm.nih.gov/geo/query/acc.cgi? acc=GSE152532).The R package used was obtained from Bioconductor (https://www.bioconductor.org/). The datasets from patients during the current study are available from the corresponding author upon reasonable request. If readers have any questions about the data processing, please do not hesitate to contact us (Qingdong Zhu: zhuqingdong2003@163.com).

## Code availability

The custom code supporting the findings of this study is available in the Supplementary material.
