# Supplementary figures and images for: A machine learning model and molecular clusters of epigenetic chromatin regulators in tuberculosis based on bioinformatics and clinical samples
Source: Sci Rep. 2025 Nov 25;15:41895. doi: 10.1038/s41598-025-25858-9 (PMC12647805; doi:10.1038/s41598-025-25858-9)

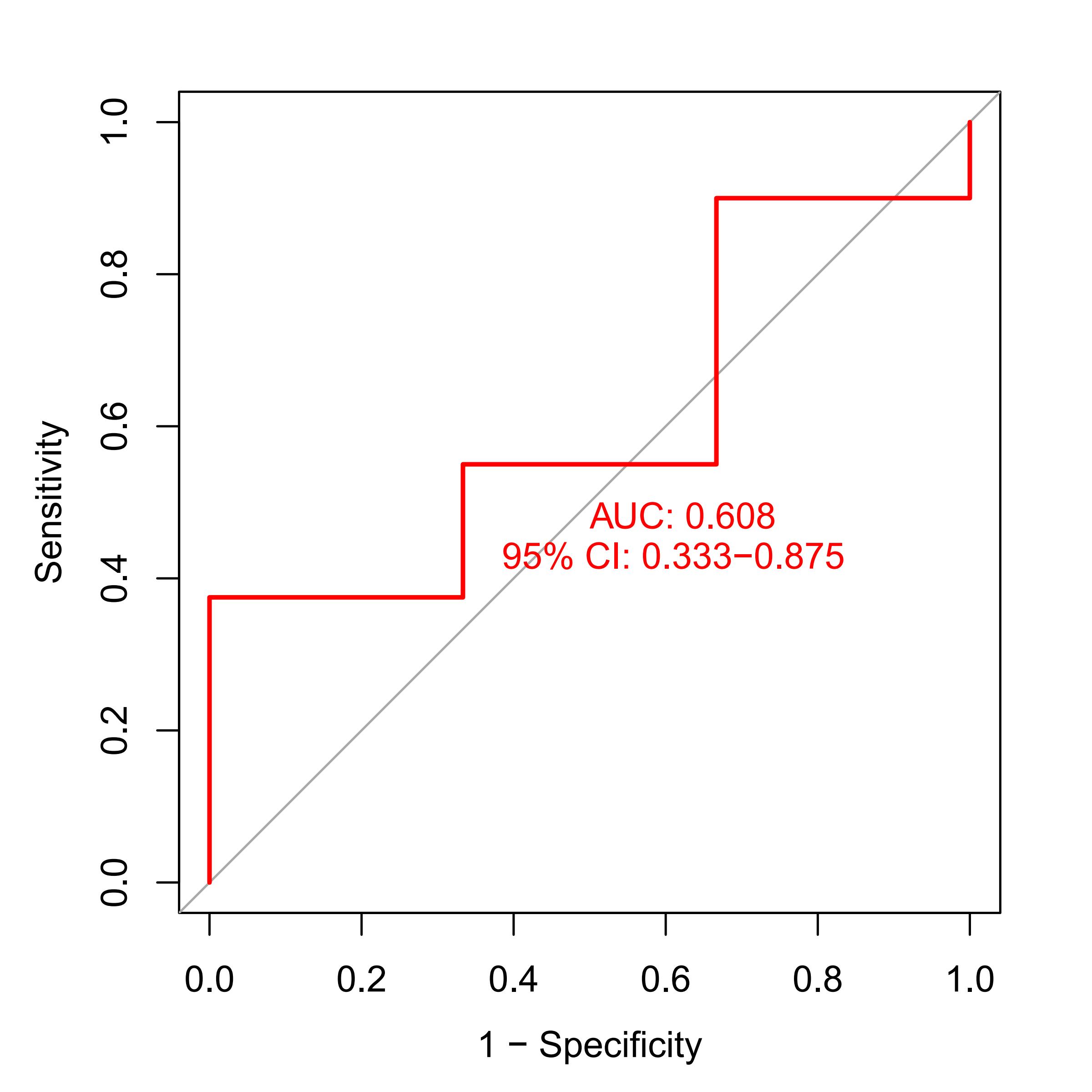

Supplement: Supplementary file 2 — Supplementary Material 2 [file 41598_2025_25858_MOESM2_ESM.jpg]
